# A social Beaufort scale to detect high winds using language in social media posts

**DOI:** 10.1038/s41598-021-82808-x

**Published:** 2021-02-11

**Authors:** Iain S. Weaver, Hywel T. P. Williams, Rudy Arthur

**Affiliations:** College of Engineering, Mathematics and Physical Sciences, University of Exeter, North Park Road, Exeter, EX4 4QF UK

**Keywords:** Environmental social sciences, Natural hazards

## Abstract

People often talk about the weather on social media, using different vocabulary to describe different conditions. Here we combine a large collection of wind-related Twitter posts (tweets) and UK Met Office wind speed observations to explore the relationship between tweet volume, tweet language and wind speeds in the UK. We find that wind speeds are experienced subjectively relative to the local baseline, so that the same absolute wind speed is reported as stronger or weaker depending on the typical weather conditions in the local area. Different linguistic tokens (words and emojis) are associated with different wind speeds. These associations can be used to create a simple text classifier to detect ‘high-wind’ tweets with reasonable accuracy; this can be used to detect high winds in a locality using only a single tweet. We also construct a ‘social Beaufort scale’ to infer wind speeds based only on the language used in tweets. Together with the classifier, this demonstrates that language alone is indicative of weather conditions, independent of tweet volume. However, the number of high-wind tweets shows a strong temporal correlation with local wind speeds, increasing the ability of a combined language-plus-volume system to successfully detect high winds. Our findings complement previous work in social sensing of weather hazards that has focused on the relationship between tweet volume and severity. These results show that impacts of wind and storms are found in how people communicate and use language, a novel dimension in understanding the social impacts of extreme weather.

## Introduction

Wind storms are responsible for significant financial losses, disruption and human casualties^1^. Due to the changing climate, their impact is expected to increase over the coming decades^2^, with high impact storms predicted to increase in frequency, particularly in Western Europe^3,4^. It is therefore important to develop robust and accurate methods for wind storm measurement, quantifying not only the likelihood, extremity and spatial extent of storms, but also their associated impacts on society. Such measurements would allow for better calibration of insurance loss and storm forecast models, as well as enabling tools for ‘now-casting’ and situation awareness^5,6^, useful for decision support and emergency response.

Over the past ten years, starting with the seminal work of^7^, a substantial literature has emerged around the use of social media, particularly Twitter, for the ‘social sensing’ of natural disasters. Here we define social sensing as observation of real-world events using unsolicited content from digital communications. Social sensing has been applied successfully for monitoring of hazards such as earthquakes^7^, wildfires^8^, floods^9,10^, pollen^11^ and air pollution^12^. Of particular relevance for this work is the application of social sensing to monitor and assess the impact of storms, hurricanes and other events associated with extreme wind^13,14^. A recent study by Spruce et al.^15^ showed that analysis of Twitter content related to named storm events in the UK and Ireland could successfully detect and track storm events over time. Tweet content was also used to assess the social impacts of the storms based on several impact categories (e.g. damage, disruption, warnings) and sentiment analysis. There is also a large related literature on the use of social media data (again, primarily Twitter) to aid response efforts during and immediately after disaster events^16–18^.

To date, social sensing has mainly used social media data in two ways. Firstly, and most commonly, the volume of event-related posts/tweets is used to infer the magnitude and/or severity of an event, based on the assumption that the number of posts is a good indicator of event size (see e.g.^8,10,11^). Secondly, the content of posts has been used to assess public mood surrounding an event. The relationship between weather (including wind and storms) and public mood has been repeatedly tested using various sentiment analysis techniques (e.g.^15,19–24^). Content analysis has also been used to extract other kinds of impact information, though such work is less common. This task requires careful interpretation of textual content, so has mostly been performed by human annotation of tweets (e.g.^15,25^), though some studies have applied automated text-mining methods (e.g.^24^). We are unaware of any studies that try to infer event size purely from the linguistic features of tweets.

Here we explore the relationship between local wind speeds and the language used in tweets. We begin by collecting tweets on wind-related keywords from 2017-04 to 2019-01, yielding a grand total of 53,442,128 tweets. Retweets are rejected (where a user re-broadcasts another user’s original tweet), automatically generated content is rejected (such as weather-station linked Twitter accounts) and tweets are narrowed down to those with a location tag within the geographic bounds of the UK, leaving 109,849 tweets to process. Our study produces a number of novel findings. After first demonstrating that linguistic features in tweets (specific words and emojis) correlate with variation in local wind speeds, we then propose a novel extension to the social sensing methodology, which seeks to quantify the severity of an observed real-world event (in this case, local wind speeds) using only the language written in social media posts, ignoring the volume of posts. The challenge throughout is to identify features of natural language written in user-generated content that are reliable indicators of high wind. The implication of this finding is that wind speed estimation can be performed in the absence of knowledge of tweet volumes. Some of the indicative words are suggestive of impacts of wind, so this study provides evidence that impact quantification by this approach is a reasonable future goal.

In spirit, this endeavour is somewhat similar to the establishment of the original Beaufort scale for wind speeds, in seeking to identify observable phenomena that correlate with particular wind speed ranges; therefore, we propose this method as a ‘social Beaufort scale’. The original Beaufort scale was devised by Sir Francis Beaufort in 1805 to regularise observations of wind speeds made by sailors, which were previously rather subjective and unreliable, by referencing a 12-point scale against visual cues for the action of the wind^26^. For example, in the modern form, Force 3 is indicated at sea by large wavelets with breaking crests, and on land, by leaves and twigs in constant motion.

The same considerations which motivated the original Beaufort scale apply here. Different users may describe the same weather conditions using different terms. One person’s ‘strong breeze’ may be another’s ‘howling gale’. There may also be regional variation, whereby severity of local winds is judged relative to the prevailing wind speeds at that location rather than against an absolute scale. There is therefore a need to calibrate language against some objective measure of weather severity. The social Beaufort scale proposed here uses a large number of tweets about wind, sampled across a range of conditions, and relates the language used in the tweet text to the local wind speed. Thereby it creates a mapping from linguistic features (words and emojis) to locally perceived wind conditions. If successful, this approach would allow social sensing to measure wind speeds without relying on the volume of tweets. Instead an accurate assessment of the wind speed might be measured based on only the words written in a few tweets. This could be useful both as an additional source of information and also in cases where rate limits and throttles are placed on the amount of data accessible from social media platforms.

We begin by describing data collection and pre-processing method, including tweet collection, filtering, and association of tweets to locally observed wind speeds. Following this, we detail our analysis, exploring the variability in wind speeds and tweet volume, as a precursor to our results, which deals with the central aim of predicting wind speeds based on tweet content. The paper concludes with a thorough discussion of the main implications of the work.

## Data collection and pre-processing

### Tweet collection and filtering

**Figure 1:**
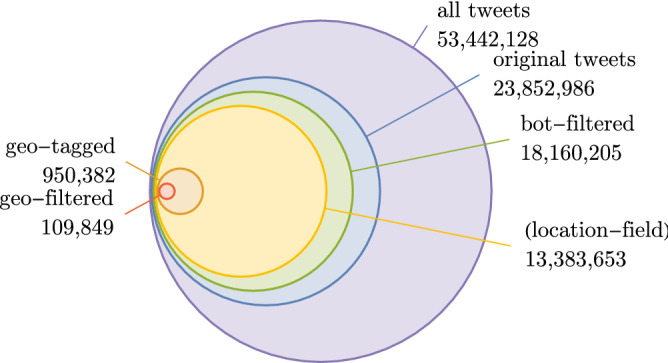
Filtering the tweet dataset. The initial dataset of of tweets containing wind-related keywords contained $\sim 53.4M$ tweets, but the majority were filtered out prior to further analysis. Filters were applied sequentially to: (i) remove retweets to leave only original tweets, (ii) remove tweets by weather stations and other bots, and (iii) retain only tweets located in the UK. After filtering, the dataset contained $\sim 109k$ tweets, approximately $0.2\%$ of the original volume.

Wind-related tweets were collected by querying the Twitter Streaming API for a set of English-language keywords: *wind*, *gale*, *windstorm*, *hurricane* and *tornado*. This yielded $\sim 53.4$ million tweets from the time period 2017-03-17 to 2019-01-01 (see Fig. 1).

This raw data set was then filtered to remove irrelevant content, using several steps:

1. *Remove retweets.* The first filter removed retweets (tweets that duplicate and re-distribute an original tweet authored by another user). Retweets are unlikely to be useful in this study since they do not represent an independent observation and may not come from the location associated with the original tweet.
2. *Remove ‘weather-bots’.* Automated social media accounts, or ‘bots’, are common on the Twitter platform and can produce a lot of content. In this dataset, manual inspection showed a large volume of tweets from amateur weather stations, whereby a weather monitoring device was linked to a Twitter account to broadcast local observations at regular intervals. Such tweets do contain weather-related information, but they do not represent human discussion of weather conditions so are not relevant for this study. Weather-bot tweets typically use a fixed template and are not written in natural language. After inspection of a large sample of such content, a weather-bot filter was implemented using a simple heuristic method that identified features common in weather-bot tweets but rare in human-authored tweets. These include specialised meteorological quantities and units (including precipitation, humidity, dew point), directions, specialised meteorological language/descriptors (such as ‘cloudless’, ‘fair’, ‘backing’), numbers and/or times. These were detected using regular expressions, as given in Table 1. Tweets containing more than six of these features were rejected, leaving $\sim 18.1$ million weather-bot filtered tweets.
3. *Location filter.* The next filter step removes tweets originating from outside the United Kingdom. This step is the most restrictive, as only $\sim 5\%$ of tweets have geographical metadata (such as a GPS coordinate, place or region name). While geographical information can be obtained using location inference^10^ based on indicators in tweet text or the user location field (approximately 13.3 million tweets have a free-text entry in the location field), here we do not use location inference due to the noise it might introduce. We retain only those tweets which are tagged as a coordinate, place or region within the United Kingdom, leaving $\sim 109,000$ tweets.

To simply test the accuracy of our bot filter, we select 1000 location-filtered tweets and manually inspect them to determine if they have been automatically generated from weather data, and compare this to our bot filter labelling. The result is displayed as a confusion matrix below. $97.3\%$ tweets which were labelled by the bot filter were in agreement with the human label. Of the $2.7\%$ where there was disagreement, $2.3\%$ of total were original tweets mistakenly labelled as bots due to an abundance of numbers and meteorological terminology, while $0.4\%$ of total were procedurally generated tweets which either used unusual abbreviations, or were very short. These results indicate that the bot filter works very well.

**Table:** 

		Manual label		Total
		Bot	Human	
Filter label	Bot	260	23	283
	Human	4	713	717
	Total	264	736	1000

The effect of applying the various filters is shown in Fig. 1. After filtering, only $\sim 109,000$ tweets are retained, representing $\sim 0.2\%$ of the volume of the initial collection.

**Table 1:** Regular expressions used to identify tweets from ‘weather-bots’. Each regex captures key terms used to describe wind and weather, including specialised meteorological vocabulary, directions and precise scientific units. Occurrence of $>6$ such tokens in a single tweet was found to be a reliable heuristic indicator that the tweet originated from a Twitter-linked amateur weather station. Tweets by weather-bots were removed from the dataset.

Regular expression	Example matches
hum(id(ity)?)?, rh	hum, humidity, rh
(in|out)?temp(erature)?	temp, outtemp, temperature
precip(itation)?	precip, precipitation
bar(o(meter)?)?	bar, baro, barometer
pres(s(ure)?)?	pres, press, pressure
dew ?p(oin)?t	dewpt, dew point
(wind[sy]?|rainy?|fog(gy)?|squally?|snowy?|clear| fair|cloud([sy]|less)?|sh(owe|w)r[ys]|backing|occ| svr|mod|gd|cool|hot|sunny|patchy|light|mild| partly|mostly)	winds, clear, cloud, cloudy, shwr
deg(rees)?|celcius|fahrenheit|[fc]	deg, f, celcius
(rising|falling)	rising, falling
(%|pct|percent)	%, pct, percent
[km]m?[p/]?(hr?|s)?	mph, m/s, mm/hr
(inhg|bar|hpa|mb|k(no)?ts?)	bar, inhg, kts
([ns]([ns]?[ew])?|[ew]([ns][ew])?)(erly)?	ne, swerly
((north|south)([$\backslash$-$\backslash$ ]?((north|south)[$\backslash$-$\backslash$ ]?)? (east|west))?|(east|west)([$\backslash$-$\backslash$ ]?(north|south)[$\backslash$-$\backslash$ ]? (east|west))?)(erly)?	northeast, south-westerly
(am|pm), [+$\backslash$-]?([01356789]|$\backslash$ d+[,/.:$\backslash$ d]*$\backslash$ d+)[+$\backslash$-]?	am, 04:20, -69.0, 10/02/2019

### Precision of tweet location

**Figure 2:**
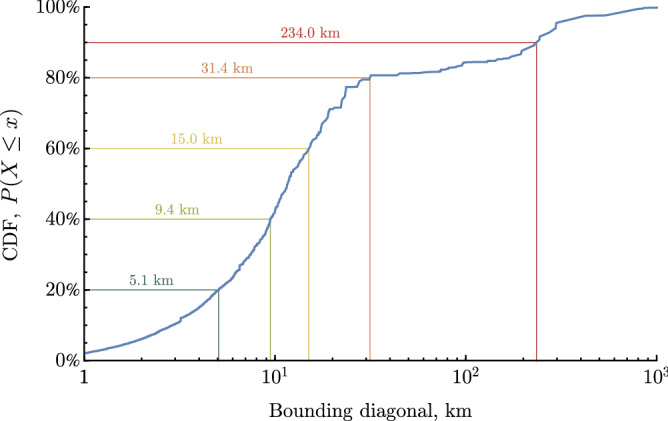
Cumulative distribution function of tweet bounding box sizes. Tweet locations are typically provided in tweet metadata as rectangular bounding boxes, measured by the diagonal length in kilometers. This plot shows the CDF for the number of corresponding tweets as box size increases. For this study, 77% of tweets are retained for further analysis by setting a maximum bounding box diagonal of 25 km.

Of the geo-filtered tweets, the vast majority are labelled with a spatial region (rather than a point coordinate); these may vary from a named road, to a town, to the entire country. Each region is represented in tweet metadata by a *bounding box*, the coordinates of a rectangle containing the region. The precision of a tweet location estimate falls quickly with increasing size of the bounding box. Here we require sufficient precision to accurately associate each tweet with a local wind speed measurement from the nearest UK Met Office weather station (see below). These stations are distributed across the UK with an average separation of 50 km. At this scale, the majority of bounding boxes in the tweet dataset are relatively small (illustrated in Fig. 2) and 77% of tweets (approximately 84*k* tweets) have a bounding box less than 25 km across the bounding box diagonal (small enough to avoid most errors in assigning the nearest weather station). We therefore set a threshold of 25 km for tweet retention and reject all tweets with larger bounding boxes. Note that this choice of 25 km is conservative; a slightly larger size of say 30 km could have been used with little change to outcomes, but above this size there is little gain and significant additional inaccuracy. Figure 2 shows an inflection in the cumulative distribution function above this threshold, meaning that even a small increase in the volume of retained tweets would require handling substantially larger regions; for example, capturing 85% of geo-filtered tweets would increase the maximum bounding box size threshold to over 170 km.

### Linking tweets to local weather observations

Weather observation data is provided by 135 weather stations throughout the United Kingdom maintained by the UK Met Office. Here we use the mean wind speed across a 20-minute interval as the wind speed observation for each station. The geographical area associated with each station is then defined using a Voronoi mesh with cells centred on each station and clipped at a maximum distance of 50 km (roughly the average distance between adjacent weather stations). The tweets retained after the filtering steps above are binned by Voronoi cell, effectively associating each tweet with its nearest weather station. Tweets that do not fall within a Voronoi cell (i.e. those tweets >50 km from a weather station) are rejected. Where a tweet bounding box does not fit entirely within a cell, the tweet is attributed to the cell with the largest area overlap.

## Variability in wind speed and tweet volume

### Spatial variability

**Figure 3:**
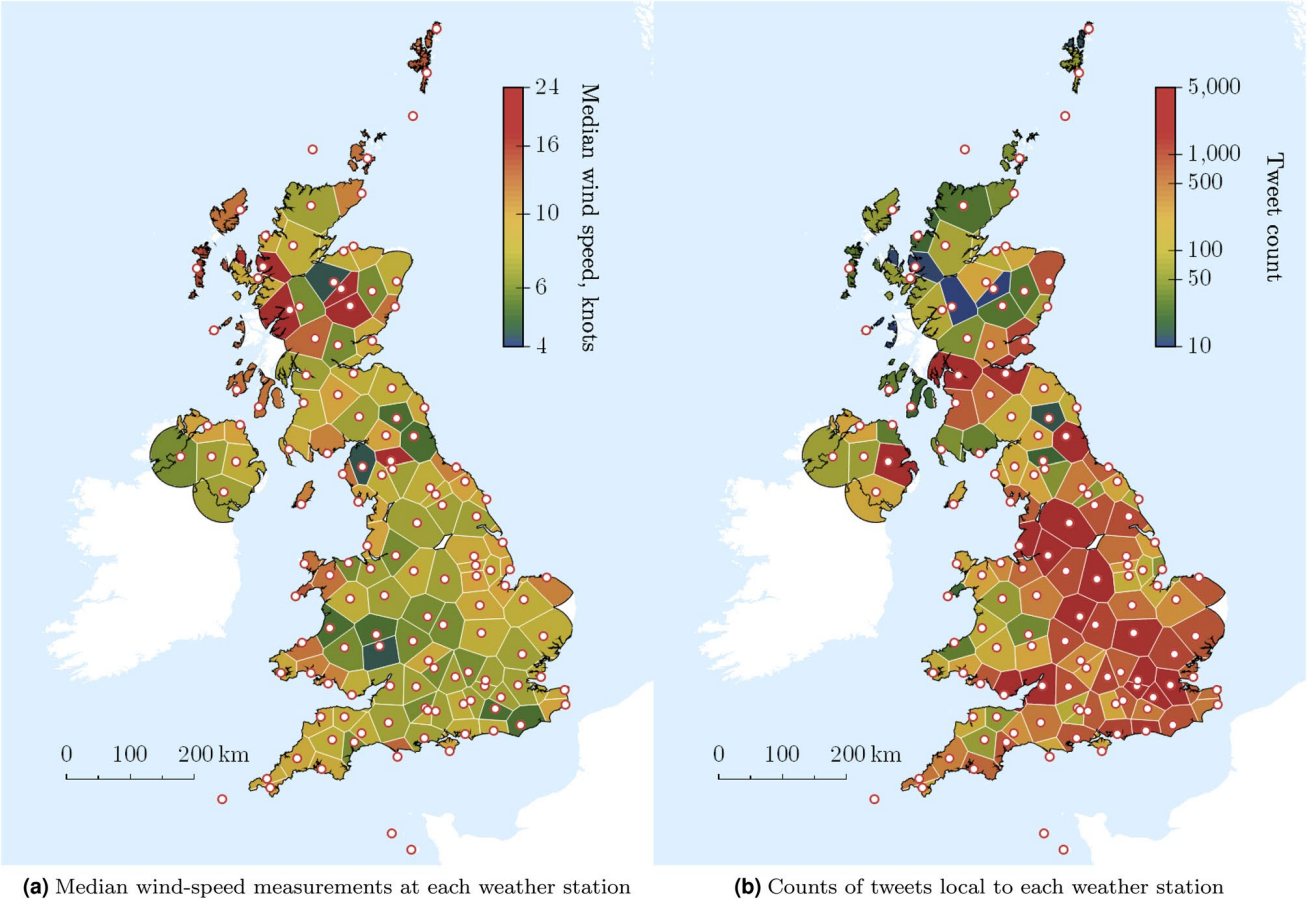
Wind speeds and tweet density across the UK. Plots show the distribution of observed wind speeds (median value of 20-minute averages across the study period) and associated tweet counts for 135 weather stations maintained by the UK Met Office. There is substantial variability in both tweet volumes (which are strongly correlated with population density) and wind speeds (with regions close to the coastline and further north generally experiencing stronger daily winds). Tweets further than 50 miles from a weather station are rejected.

Figure 3a shows the location of the 135 weather stations, with median wind speeds for each station during the study period shown as a heatmap. Figure 3b shows the total count of geo-filtered tweets in the Voronoi cell surrounding each weather station. Both local wind speeds and associated tweet counts show substantial spatial diversity across the UK. Tweet counts are (as expected) greater in areas with greater population density. Wind speeds are higher in coastal and northern regions.

### Temporal variability in tweet volume

**Figure 4:**
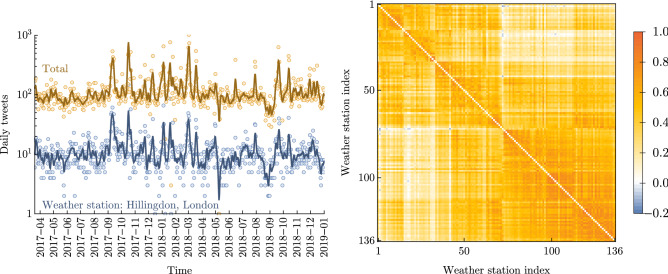
Local-national correlations in tweet volumes and wind speeds. *Left panel*: Daily tweet volumes for the whole UK (total number of tweets within 50 km of any UK Met Office weather station; yellow) and local to the single highest-volume weather station (Northolt, London; blue). Points show daily counts while the 7-day moving average is shown with solid lines. There is substantial temporal variability in both time series. The two time series are strongly correlated (see text). *Right panel:* Pairwise Spearman’s rank correlations for wind speed timeseries for all weather stations. Strong positive correlations are seen for almost all weather station pairs, though the strength the correlation is variable. This indicates a high level of local-national correlation in wind speeds.

Figure 4 (left panel) shows daily tweet counts summed across all cells (i.e. the whole of the UK) during the study period, alongside daily tweet counts for the highest-volume cell (centred on the weather observation station in Northolt, London). There is order-of-magnitude variation in daily tweet counts for both time series. The two time series are very strongly correlated with large spikes of activity mirrored locally and nationally (Pearson correlation $r=0.85,p<0.0001$). Similarly high temporal correlations are found for other local regions (data not shown) suggesting that volumes of local and national Twitter activity related to wind are strongly correlated in general.

In the simplest case, Twitter activity and local wind speeds are unrelated, with Twitter activity instead driven by attention to tangential topics with keywords caught in our Twitter collection, such as *wind farms*. However, it is plausible that wind-related Twitter activity is driven at least in part by local or national weather conditions, so we might expect the two datasets to be related. This might occur in two ways: (i) people tweet in response to local wind speeds, with correlation between local and national wind speeds leading to correlation in local and national tweet volumes; or (ii) people tweet in response to (social or traditional) media coverage of large scale wind events. Both mechanisms might operate at different times. In both cases, pre-emptive or delayed Twitter activity can provide an obfuscating mechanism, where tweets are expected to lead or lag behind local or national weather conditions. In particular, the dataset includes several named storms including hurricane Ophelia which was well covered in the media prior to hitting the UK. Figure 4 (right panel) shows that wind speeds are indeed correlated across the UK. This evidence, together with manual inspection of tweets, suggests that the first mechanism may be dominant; local and national wind speeds are correlated, so local and national tweet volumes are correlated.

## Predicting wind speeds with linguistic features in tweets

In this section, the aim is to establish the extent to which wind speeds can be estimated from tweet content, i.e. to identify linguistic features (words, emojis) that indicate high winds.

### Linking wind speeds to tweets

Before we can try to identify the components of a tweet message that are indicative of the local wind speed, we first need to associate a wind speed with each tweet. There are several ways in which this can be done. The naive approach would simply attach the absolute local wind speed to each tweet, assuming that people respond to the absolute wind speed (and by implication, that people everywhere have the same response to wind speed). However, looking at Fig. 3a, we can see that there is substantial variation in prevailing wind speeds across the UK. Previous work^27^ demonstrates how Twitter users’ response to flooding relates to the ‘remarkability’ of such events. Similarly, it is reasonable for us to expect that a person’s expectations of the weather may affect how they perceive a given absolute wind speed and alter their (linguistic) response. For example, an absolute wind speed that would be remarkable in London, and trigger particularly emphatic language from London-based users, might be perceived as unexceptional and generate no comment in North West Scotland, where wind speeds are frequently high. For this reason, it may be beneficial to perform a transformation such that tweets are associated with wind speeds normalised to local conditions (i.e. associate tweets to winds that are *locally* high or low).

**Figure 5:**
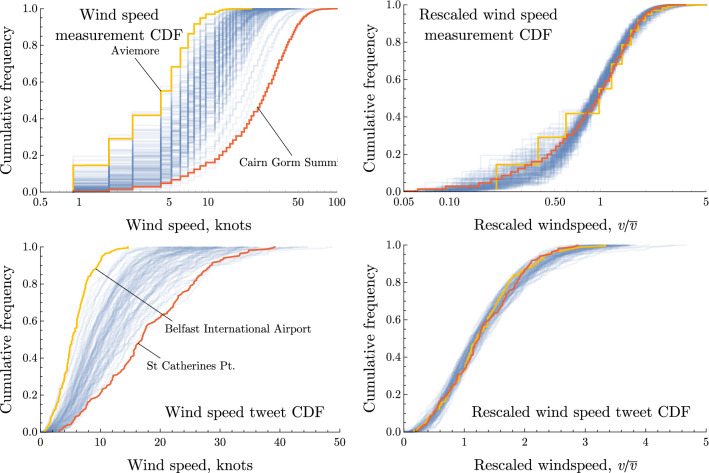
Local re-scaling of wind speeds. *Top panels*: Cumulative distributions of wind speeds at each weather station, with the stations having highest and lowest mean wind speeds highlighted. In the left panel, the data is unaltered. In the right panel, each distribution has been re-scaled by dividing each wind speed by the mean value for the distribution. *Bottom panels*: Cumulative distributions of wind speeds associated with individual tweets. In the left panel, wind speeds are unaltered. In the right panel, each distribution is re-scaled by dividing each wind speed by the mean value for the distribution.

Figure 5 shows the cumulative distributions of wind speed measurements (top), along with cumulative distributions of wind speeds associated with tweets (bottom), at each weather station. Left panels show absolute local wind speeds measured in knots. Right panels show local wind speeds transformed by a simple linear re-scaling. Both distributions undergo the same transformation; each wind speed value *v* is divided by the mean wind speed $\overline{v}$ at that weather station. Un-scaled wind speeds show distributions which are similar in shape for all weather stations, but vary in their mean value. Re-scaling causes the distributions to converge to a great extent, showing that normalising by the local mean wind speed removes the main differences between geographic areas in terms of experienced wind speeds. This strongly implies that the Twitter response to changes in local wind speeds is a function of local average conditions; that is, users respond to deviations from the local expectation of prevailing winds. In both cases, the resulting distributions have a near-perfect fit to a gamma distribution, for which the probability density function is given by $P(x)=\frac{x^{k-1}e^{-\frac{x}{\theta }}}{\theta ^k\Gamma \left( k \right) }$. For re-scaled wind speeds, best fit shape *k* and scale $\theta$ parameters are $k=3.1,\theta =\frac{1}{k}$, while for re-scaled tweet volumes the equivalent values are $k=3.31,\theta =0.40$, with means $\mu =k\theta =1$ and 1.31 respectively. For all subsequent analysis, we proceed by labelling tweets with the locally re-scaled wind speeds to normalise this regional variation.

### Linking linguistic tokens to wind speeds

In order to study elements of tweet text which correlate with windy weather, we next need to associate every token in tweet text (words, emojis) with the set of wind speeds in which they were generated. This step uses the locally re-scaled wind speeds described in the previous section, to normalise for regional variation in typical wind speeds experienced by users.

Tweet text is tokenised by breaking on spaces and punctuation. The words are then stemmed (using an English-language snowball stemmer implemented by the Python Natural Language Toolkit *nltk* (https://www.nltk.org/)), so that inflected words are reduced to their ‘stem’ e.g. ‘howling’ and ‘howled’ would be reduced to ‘howl’. Stemming is useful here to reduce the number of tokens under consideration, since many words with different tense or plurality have the same meaning. Although there are instances where language is not so simple, such as ‘freezing’ and ‘frozen’ which have different stems, it remains an efficient and useful tool for this purpose. Stems are recorded so that stemmed tokens can later be replaced with their most common un-stemmed counterparts for visualisation. After stemming, tokens which appear in less than 0.5% of tweets are removed from further analysis; in the current dataset, this threshold ensures that retained tokens appear in at least $\sim 500$ tweets. This threshold is a pragmatic choice that balances retention of tokens against exclusion of very rare ‘noise’ tokens such as typos and mis-spellings. Rarer tokens are more likely to correlate with high- or low-wind conditions *by chance*, which can strongly contribute to classifiers *over-fitting*; we discuss over-fitting shortly.

**Figure 6:**
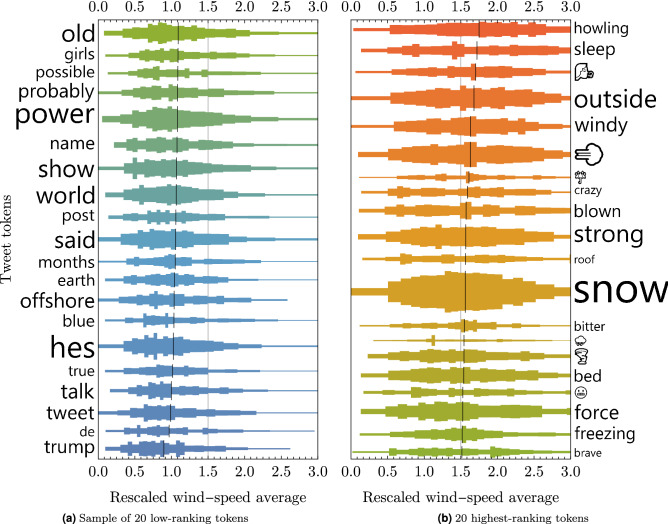
Distributions of wind speeds associated with a selection of words and emojis. Wind speed values are average wind speeds at the nearest weather station in the 6 hours prior to each tweet’s creation. Distributions are compiled from the set of all tweets containing each token.

Next, distributions of locally re-scaled wind speeds were created for each stemmed token, based on the wind speeds associated with all tweets that contained them. Tokens were then ranked based on the mean value of their wind speed distribution. Distributions for the 20 highest-ranking tokens and a sample of 20 low-ranking tokens are shown in Fig. 6. Distributions for the low-ranking tokens are associated with average wind speeds, with values clustered around 1 (i.e. the mean value after re-scaling) and relatively small variance. In contrast, high-ranking tokens are more loosely distributed, with greater variance, but have mean values more than 50$\%$ higher than local average wind speeds.

These patterns show several interesting features. Firstly, low-ranking tokens in wind-related tweets are not associated with low winds, but instead co-occur with average wind speeds. This suggests that there is a minimum wind speed threshold for *any* wind-related tweets to be generated. Secondly, high-ranking tokens typically co-occur with above-average wind speeds, but are used across a wide range of wind speeds. Finally, there is an intuitively reasonable semantic coherence to the high-ranking tokens, which are mostly words related to wind and its effects, whereas the low-ranking tokens are semantically un-related to wind. Interestingly, emojis are disproportionately common in the top-20 high-ranking tokens.

### Classifying ‘high-wind’ tweets based on linguistic features

The previous section demonstrated associations between individual word/emoji tokens and wind speeds. This suggests that whole tweets can be classified as relating to either high or average wind speeds based on the set of tokens they contain. Reliable classification would indicate that linguistic tokens alone are sufficient to infer wind speeds. Classification can also be used to filter out average-wind tweets, to test whether focusing on high-wind tweets improves the ability to predict local wind speeds using tweet volumes.

The first step in this process is to build a classifier to separate ‘high-wind’ tweets from ‘average-wind’ tweets, based on the set of stemmed tokens that each tweet contains. The findings shown in Fig. 6 suggest that we will not be able to use this dataset to identify ‘no-wind’ or ‘low-wind’ tweets, since the lowest-ranked tokens are associated with average wind speeds; the presence of any wind-related tweets indicates that wind speeds are at or above the local average. This reflects the data collection methods, which began by collecting tweets that utilised one of several wind-related keywords.

A decision tree classifier was constructed to predict whether a tweet is labelled with a local wind speed in the top quartile of local wind speeds based on the set of stemmed tokens (words and emojis) it contains. It is important to avoid over-fitting this filter; while we have ensured all tokens are well represented in our data set, combinations of specific tokens are exponentially rarer and a decision tree may find combinations which correlate to high wind speeds by chance, in spite of an absence of correlation independently. The specific implementation of our classifier is found to be largely unimportant in this study, with decision trees, random forests and regression models producing similar results. In the following, we present methods and results for a different classifiers implemented using the popular *scikit-learn* Python package (https://scikit-learn.org/).

The classifier was trained on a random sample of half the tweet dataset and then tested on the remainder, both $N=39,334$. The Gini impurity was used as splitting criterion with the best feature used to split at each branch. Due to a large number of low-frequency tokens, the decision tree model will tend to over-fit training data, producing misleading results although this can be combated by limiting the tree depth. Generally speaking, we find increasing tree depth to result in lower precision and higher recall with a slight overall increase in F1 score and decrease in accuracy. On the other hand, increasing the number of text features utilised in trees beyond a couple of dozen quickly over-fits the training partition, resulting in a reduction in all performance metrics. Table 2 shows the precision, recall and accuracy of several methods, where parameters have been adjusted in each case to maximise accuracy. The confusion matrix for the random forest performance on training data is shown in detail by Table 3. Overall accuracy is 0.65, compared to a naive accuracy of 0.51 that could be achieved by always choosing the most frequent label (in this case ‘negative’).

**Table 2:** Four different classifiers are trained on a sample of 50% of the filtered and labelled dataset, and then we test their performance on the other 50% of the data across four metrics. In each instance the features used (*f*) and tree depth (*d*) are selected to maximise accuracy.

	Precision	Recall	F1	Accuracy
Linear regression	0.60	0.53	0.57	0.60
Decision tree ($f=28, d=52$)	0.62	0.39	0.48	0.58
Random forest ($f=26, d=50$)	0.65	0.45	0.53	0.61
Random forest regressor ($f=28, d=52$)	0.64	0.46	0.54	0.61

**Table 3:** Random forest classifier performance on test data, having been trained on an equally sized partition of training data. The high false negative rate of predictions is largely due to tweets which do not contain relevant tokens in spite of local weather conditions. In contrast, relatively few tweets contain relevant tokens without corresponding local winds.

		True label		
		Positive	Negative	Total
Predicted label	Positive	11,077	1646	12,723
	Negative	8377	18,234	26,611
	Total	19,880	19,454	39,334

While performance of the classifier may seem modest compared to some other machine learning tasks, it must be remembered that this is a fundamentally difficult classification task. An individual tweet contains few tokens and most tokens indicating high-wind conditions are also used in more moderate wind conditions, so there is no clean separation of positive and negative cases. The high precision is encouraging for the purposes of this study. It suggests that this classifier could read a *single* tweet and identify relatively high-wind conditions, being correct in 65% of cases. If multiple tweets were used, accuracy could be greatly improved.

### A simple Beaufort scale using linguistic features to indicate wind speeds

**Figure 7:**
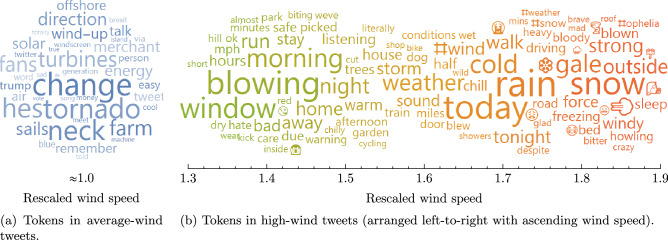
Linguistic tokens ordered by wind speed. Tokens are positioned left-to-right by increasing wind speed (interpreted as the median value from the distribution of locally rescaled wind speeds associated with tweets containing the token). The size of each token indicates the frequency of its use in wind-related tweets. Only the most commonly used tokens are shown. The token ‘tornado’ is associated with average wind speeds because tornadoes are extremely rare in the UK and these tweets are invariably discussing tornadoes in other countries.

Summarising the wind speed distributions for each token by their median value, another ranking can be used as a scale to indicate local wind speeds by the words/emojis used in tweets. Figure 7 shows an expanded set of wind-related tokens, sorted by the median value from the distribution of wind speeds associated with the tweets in which they appear. High-wind tokens include a variety of wind-themed words (e.g. ‘blown’, ‘gale’, ‘howling’) and emojis (icons representing wind, tornadoes, etc.). Also amongst the high-wind tokens are other weather conditions which might co-occur with wind (e.g. ‘snow’, ‘rain’) and words suggestive of impacts of wind (e.g. ‘sleep’, ‘roof’). Amongst the tokens that are associated with average wind speeds are words related to wind energy (e.g. ‘offshore’, ‘farm’, ‘turbines’) as well as ‘wind-up’ (a colloquial phrase meaning to tease or provoke).

The ranking in Fig. 7 serves as a scale with which wind speeds (measured relative to local prevailing conditions) can be inferred from the linguistic tokens (words, emojis) being used in tweets produced near a weather station. This methodology is analogous to the original Beaufort scale mapping absolute wind speeds against visual cues that can be seen in the surrounding environment. However, this ‘social Beaufort scale’ differs in that it is localised; linguistic features indicate high winds relative to local conditions. That is, the words and emojis are indicative of relatively high wind speeds after local re-scaling, rather than absolute wind speed measurements. These contextual aspects mean that the social Beaufort scale can be used to determine whether it is currently unusually windy at a given location, but cannot be used to measure actual wind speeds on a quantitative scale.

### Correlation between ‘high-wind’ tweet frequency and local wind speeds

**Figure 8:**
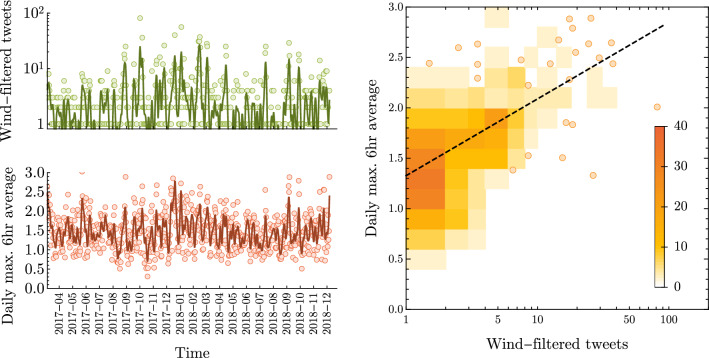
Correlation between ‘high-wind’ tweet frequency and local wind speeds for the weather station at Northolt, London. *Left panel:* Daily counts of filtered high-wind tweets compared with daily maximum wind gust. Timeseries plots give 7-day moving averages (solid line) and daily values (points). The two time series have Pearson’s correlation of $r=0.57$. *Right panel:* Density of days with different wind speeds and tweet counts, where points are shown where the bin count would be one. Plot shows a strong positive association between wind speed and tweet volume.

We now test the correlation between the frequency of high-wind tweets (classified as above) and local wind speeds. As an example case, Fig. 8 (left panel) plots the daily count of (filtered) high-wind tweets and daily maximum 6-hour average wind speed over time, for the weather station with the highest total tweet count (Northolt, London). Recall that tweets are labelled by the average wind speed in the 6 hours prior to the tweet being published. There is a significant relationship between high-wind tweet volume and local wind speed, characterised by a Pearson correlation statistic of $r=0.57$.

Figure 8 (right panel) shows the relationship between high-wind tweet volumes and wind speeds by plotting the density of days for pairwise combinations of tweet volume and wind speed. A strong positive relationship can be seen, with every day on which >3 tweets were recorded showing greater than average wind speeds.

The relationship seen at Northolt weather station is replicated widely across the UK. Table 4 gives Pearson’s *r* correlation values for the top-10 (strongest correlation) and bottom-10 (weakest correlation) weather stations in the dataset. Data is shown for unfiltered (average-wind or high-wind) tweets and filtered (high-wind) tweets. In the absence of filtering, correlations between tweet volumes and local wind speeds are weak (range $r=0.18-0.36$), but after filtering the correlations range from modest to strong (range $r=0.25-0.57$). Unsurprisingly, the best performing sites are generally (though not strictly) those with a large corresponding tweet count.

**Table 4:** Correlation between tweet volume and local wind speeds at a selection of UK weather stations. Data shown: Pearson’s correlation coefficient, *r*, for relationship between daily tweet counts and mean wind speeds for all tweets. Data are given for both unfiltered (average-wind or high-wind) and filtered (high-wind) tweets. Top-10 (strongest correlation) and bottom-10 (weakest correlation) stations are given.

Station name	Tweet count	r (unfiltered)	p-value	r (filtered)	p-value
Northolt	7380	0.25	$10^{-10}$	0.57	$10^{-57}$
Bingley Samos	3564	0.36	$10^{-21}$	0.56	$10^{-54}$
Crosby	3312	0.28	$10^{-13}$	0.54	$10^{-50}$
Edinburgh/Gogarbank	2907	0.34	$10^{-19}$	0.54	$10^{-49}$
Kenley	3650	0.27	$10^{-12}$	0.53	$10^{-48}$
Watnall	3236	0.27	$10^{-12}$	0.51	$10^{-44}$
Filton	1927	0.24	$10^{-9}$	0.51	$10^{-39}$
Gravesend-Broadness	2582	0.21	$10^{-6}$	0.51	$10^{-34}$
Belfast International Airport	1678	0.30	$10^{-14}$	0.51	$10^{-43}$
Rostherne No 2	3535	0.28	$10^{-13}$	0.50	$10^{-42}$
$\vdots$	$\vdots$	$\vdots$	$\vdots$	$\vdots$	$\vdots$
Benson	960	0.20	$10^{-7}$	0.31	$10^{-15}$
Shoeburyness	611	0.21	$10^{-8}$	0.30	$10^{-15}$
Wattisham	737	0.19	$10^{-6}$	0.30	$10^{-14}$
Heathrow	1759	0.21	$10^{-7}$	0.28	$10^{-13}$
Linton On Ouse	717	0.25	$10^{-10}$	0.27	$10^{-12}$
Leek	803	0.18	$10^{-6}$	0.26	$10^{-11}$
Church Lawford	945	0.21	$10^{-8}$	0.26	$10^{-11}$
Aberdeen Airport	705	0.20	$10^{-7}$	0.25	$10^{-10}$
Prestwick Rnas	650	0.20	$10^{-7}$	0.25	$10^{-10}$
Hawarden	504	0.25	$10^{-10}$	0.25	$10^{-10}$
Farnborough	646	0.18	$10^{-6}$	0.25	$10^{-10}$

## Discussion

In this paper, we set out to determine whether the language and vocabulary used in tweets relating to wind could be used to develop a robust method for measuring wind speeds. Towards this aim, we began by collecting a large dataset of tweets including wind-related keywords (*wind, gale, windstorm, hurricane, tornado*) and filtering to retain only original tweets by human users in the UK. Each tweet was then associated with a local wind speed based on a Voronoi-mesh binning procedure with cells centred on 135 UK Met Office weather stations. Initial analysis showed spatial variation in wind speeds (with higher speeds seen in northern and coastal areas) and tweet volume (with volumes proportional to population density). The next step was to control for this variation by applying a simple re-scaling transformation to absolute wind speeds associated with tweets, normalising wind speeds by local average conditions. This permitted a distribution of local wind speed values to be associated with each linguistic token (word or emoji) in tweet texts. A set of tokens was found that are robustly associated with high winds relative to local conditions; conversely, a set of tokens was found whose occurrence is associated with average (and unexceptional) wind speeds. The high-wind tokens were used in multiple ways to recover wind speeds solely from Twitter data.

The first approach to inferring wind speeds from Twitter data takes advantage of the association between linguistic tokens and wind speeds. A decision tree classifier was developed to detect tweets associated with high winds. Here the classifier was used as a filter to partition tweets relating to high winds from those captured in the data collection for other reasons. Interestingly, tokens indicating calm or low-wind conditions seem not to be captured by wind-related search terms. It is possible that no such tweets exist. Experience with Twitter data suggests that tweets tend to contain news-worthy information or comment on an unfolding situation; a tweet reporting a lack of wind would seem unusual by this convention, as it essentially reports a lack of an event. Instead, the lowest-ranked tweets and tokens found here were associated with average wind speeds relative to local condition, and tended to use a wind-related phrase that does not relate to immediate weather conditions (e.g.‘windfarm’, ‘wind-up’). Thus they are independent of wind speeds, rather than reporting on moderate winds. The classifier developed here showed reasonable performance, with recall of 0.45 and precision of 0.65. The recall score is quite conservative, suggesting that many high-wind tweets would not be detected. However, the precision score is quite high, suggesting that when the classifier identifies a high-wind tweet, there is good likelihood that local wind speeds are high. This finding suggests this methodology could be employed to detect high winds from only a single tweet at a given location.

The second approach to using linguistic features of tweets to determine wind speeds is analogous in spirit to an attempt to create a ‘social Beaufort scale’, mapping words and emojis to local wind conditions. This attempt was partially successful. Taking the median value from the distribution of wind speeds associated with tweets containing each token, where wind speeds are interpreted as values re-scaled by locally prevailing wind conditions, a ranking was produced that maps individual tokens to an expected wind speed. This is similar to the original Beaufort scale, but has several important differences: firstly, the social Beaufort scale works with locally re-scaled wind speeds rather than absolute values, so it does not give an indication of the wind speed in standard units; secondly, it measures the experienced wind speed relative to local conditions, so does not provide a context-free measurement. It would be possible to produce a similar social Beaufort scale tuned to a particular location (e.g. a single weather station) that could predict absolute wind speeds, by omitting the local re-scaling step in the methods above. However, this ability would come at the cost of generality, with a separate scale needed for each location. As reported here, the social Beaufort scale does provide evidence that language alone can be used to infer wind speeds, and does so in a way that corrects for spatial variation in prevailing conditions. This captures an important aspect of wind as experienced by people; in normally calm places, strong winds may be remarkable, whereas the same wind speed would be considered typical in windier places such as northern and coastal areas.

Utilising the ability to identify high-wind tweets, a third approach to mapping wind speeds from Twitter data was demonstrated. The high-wind filter was applied to all tweets and the number of filtered tweets in 24-hour blocks was correlated against local wind conditions. Most regions showed strong correlations between (filtered) tweet volume and wind speeds. This approach is most similar to volume-based social sensing methods that have been applied elsewhere (e.g.^10,11,15^).

A limitation of this work relates to data collection. A keyword search was used to retrieve tweets from the Twitter API. Collecting in this way introduces biases by limiting the sample of tweets that are examined in this study. We cannot, for example, claim generalities around Twitter users’ weather communications, only examine those which also contain our keywords. Future work can improve upon this aspect, such as by collecting all geotagged tweets within the relevant bounding box and thereby removing dependence on keywords. Such a collection will be overwhelmingly weather agnostic, but would provide a broader linguistic foundation for a more thorough study. This said, we have confidence that the qualitative findings of this manuscript are robust to most of our design decisions and would only be strengthened by a more thorough treatment at any stage of the investigation.

As another future consideration^27,28^, consider temporal variability in the remarkability of extreme weather events, particularly in the context of rapid climate change. There is evidence that climate change can have a significant on wind speed^29^, although it is unlikely to be significant over the course the 20-month time span in this study. This means that the linguistic features that are associated with high winds may change over time, as the population producing language instances becomes more or less sensitised to high winds; today’s high wind may be tomorrow’s light breeze. The evolution of language may itself change the words and tokens used to indicate severe or newsworthy wind, adding a further temporal dimension.

The social Beaufort scale introduced here suggests that the relationship between linguistic features (words and emojis) and locally perceived wind conditions might be successfully used to allow social sensing to measure wind speeds without relying on the volume of tweets. Instead an accurate assessment of the wind speed can be measured using only the words written in a single tweet, with improvements likely to be possible if multiple tweets are combined. This volume-independent approach has the potential to greatly improve now-casting and situational awareness, and provide a computationally cheap and efficient method for wind speed estimation. Such methods may also allow social sensing software and architectures to be more robust to changes in social media data provision (e.g. the Twitter API changes occasionally without documentation^30^), as well as variation in the popularity of platforms over time and in different places. The approach has some similarities with sentiment analysis^31^, in which individual words are assigned a positive/negative valence. However, the approach here is correlative, starting from the observed vocabulary associated with observed wind speeds. It might permit a valence dictionary to be created for the weather domain in future work and the methodology described here could be applied to other weather types with variable magnitude, e.g. rain, snow, heat, fog.

In summary, this paper has shown that social sensing can be used to provide measurement of wind, either using tweet volume alongside tweet content, or using tweet content alone (cf. the social Beaufort scale). Content-aware methods offer new approach to social sensing, which typically uses the message content only to filter for relevance prior to quantification using tweet volumes. The work presented here helps to give an indication of people’s subjective experience of weather, and is a precursor to study of impacts and social responses to wind as revealed by language. Such observations can be very difficult to capture otherwise and can be useful for measuring the social impact of weather events. The meteorological community is increasingly turning to impact-based forecasting methods, which produce warnings and alerts based not just on the likelihood of a weather event, but on the event’s likely impact on human health, well-being and normal activity. Social sensing in general, and content-based methods in particular, may help to provide essential validation data for such approaches.
